# Underlying Small Vessel Disease Associated With Mixed Cerebral Microbleeds

**DOI:** 10.3389/fneur.2019.01126

**Published:** 2019-10-23

**Authors:** Clemence Blanc, Alain Viguier, Lionel Calviere, Mélanie Planton, Jean François Albucher, Vanessa Rousseau, Agnès Sommet, Fabrice Bonneville, Jérémie Pariente, Jean Marc Olivot, Nicolas Raposo

**Affiliations:** ^1^Neurology Department, Hôpital Pierre-Paul Riquet, Centre Hospitalier Universitaire de Toulouse, Toulouse, France; ^2^Toulouse NeuroImaging Center, Université de Toulouse, Inserm, UPS, Toulouse, France; ^3^Epidemiology Department, Centre Hospitalier Universitaire de Toulouse, Toulouse, France; ^4^Department of Clinical Pharmacology, CIC1436, USMR, Centre Hospitalier Universitaire de Toulouse, Toulouse, France; ^5^Neuroradiology Department, Hôpital Pierre-Paul Riquet, Centre Hospitalier Universitaire de Toulouse, Toulouse, France

**Keywords:** intracerebral hemorrhage, cerebral amyloid angiopathy, cerebral microbleeds, cortical superficial siderosis, cerebral small vessel disease, neuroimaging

## Abstract

**Background and Purpose:** Whether patients with both lobar and deep cerebral microbleeds (mixed CMB) have advanced cerebral amyloid angiopathy (CAA), hypertensive angiopathy (HA) or both is uncertain. To get insight into the underlying small vessel disease (SVD) associated with mixed CMB, we explored its association with cortical superficial siderosis (cSS), a key marker of CAA and other MRI markers of SVD in patients with intracerebral hemorrhage (ICH).

**Methods:** Of 425 consecutive patients with acute ICH who had received brain MRIs, 260 had ≥1 CMB and were included in the analysis. They were categorized as strictly lobar CMB (suggesting CAA), strictly deep CMB (suggesting HA) or mixed CMB. Clinical and imaging characteristics were compared (1) between the three CMB groups and (2) within mixed CMB patients according to the symptomatic ICH location.

**Results:** Overall, 111 (26%) patients had mixed CMB. Compared to strictly lobar CMB (*n* = 111) and strictly deep CMB (*n* = 38), patients with mixed CMB had a more severe burden of lacune, white matter hyperintensities and CMB. cSS was observed in 24.3% of patients with mixed CMB compared to 44.1% in strictly lobar CMB and 10.5% in strictly deep CMB (*p* < 0.0001). Among patients with mixed CMB, 44 (39.6%) had a lobar symptomatic ICH and 67 (60.4%) had a non-lobar ICH. Patients with non-lobar ICH were more likely to have hypertension, whereas those with lobar ICH were more likely to have cSS and chronic lobar ICH and had higher ratio lobar CMB count/total CMB count.

**Conclusions:** Mixed CMB is frequently encountered in patients with ICH and appears as a heterogeneous group, suggesting that both CAA and HA may be contributing to mixed CMB. Neuroimaging markers including ICH location, cSS, and CMB distribution may indicate the predominant underlying vasculopathy, with potential prognostic implications.

## Introduction

Hypertensive angiopathy (HA) and cerebral amyloid angiopathy (CAA) are common small vessel diseases (SVDs) that account for most cases of spontaneous intracerebral hemorrhage (ICH) (1), the most severe stroke subtype with high risk of mortality and dementia (2, 3). CAA results from amyloid-β (Aβ) deposition in the wall of cortical and leptomeningeal vessels and is a common cause of lobar ICH. By contrast, HA is characterized by lipohyalinosis, arteriolosclerosis, and fibrinoid necrosis, predominantly affecting deep perforating arteries, causing deep or infratentorial ICH (4).

Cerebral microbleeds (CMBs) are neuroimaging markers of SVD detected in ~60% of patients with spontaneous ICH (5). The pattern of CMB distribution is suggestive of the underlying SVD (6). Patients with multiple CMB confined to the lobar regions are likely to have CAA (7). Conversely, CMBs are typically located in the deep regions (basal ganglia, thalamus, and brainstem) in HA (8). When patients present with both lobar and deep CMB (i.e., mixed CMB), it is unclear whether they have advanced HA, CAA, or a combination of these two diseases. Understanding the underlying SVD has important clinical implication with regard to the risk of ICH recurrence, anticoagulant strategy, and patient selection for anti-amyloid therapy trials.

In the current study, we aimed to explore the underlying SVD(s) associated with mixed CMB in patients with symptomatic ICH. We investigated the clinical and imaging factors associated with mixed CMB compared to strictly lobar CMB (suggesting CAA) and strictly deep CMB (suggesting HA). We also evaluated whether CMB distribution in patients with mixed CMB may indicate underlying SVD by testing the associations of the ratio lobar / total CMB count with symptomatic lobar ICH and cortical superficial siderosis (cSS), key neuroimaging markers of CAA (7, 9, 10).

## Materials and Methods

The data that support the findings of this study are available from the corresponding author on reasonable request.

### Patient Selection

We conducted a retrospective cross-sectional analysis of data collected prospectively from consecutive patients admitted between December 2011 and January 2016 to Toulouse Hospital stroke center for acute spontaneous ICH who underwent brain MRI. The study was approved by the Toulouse-Purpan Hospital Research Ethics Committee (No. 22-0315). Informed consent was not required because of the retrospective observational design of the study.

Inclusion criteria for this study were as follows: (1) patient with acute spontaneous ICH, (2) MRI of adequate quality performed within 30 days after symptom onset (3) presence of ≥ 1 CMB. Patients with traumatic ICH, secondary causes of ICH, and those without CMB were excluded. To limit diagnostic uncertainty, we also excluded patients with CMB restricted to the cerebellum.

### Data Collection

Demographic and clinical data (age, sex, vascular risk factors, anticoagulant, history of symptomatic ICH) were recorded and analyzed. Information on neurological symptoms upon admission was collected.

### MRI Acquisition and Analysis

MR images were acquired on 1.5-T or 3-T scanners and included at least T2^*^-weighted gradient recalled echo (T2^*^-GRE) and axial fluid-attenuated inversion recovery (FLAIR) sequences, as previously described (11). Among the 260 patients with CMB included in the analysis, 148 (56.9%) underwent a 3-T MRI and 112 (43.1%) a 1.5-T MRI. Among patients with mixed CMB, the proportion of participants who underwent a 3-T MRI (61.3%) was not statistically different from patients with strictly lobar CMB (55.9%; *p* = 0.45) or strictly deep CMB (47.4%; *p* = 0.13). CMB count was similar between patients with 3-T MRI and those with 1.5-T MRI in each CMB group.

**Figure 1:**
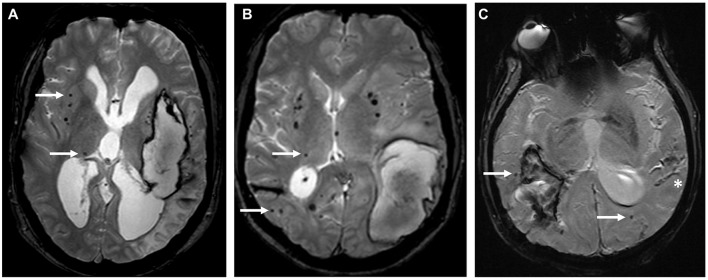
Different distribution patterns of cerebral microbleeds. T2*-GRE MRIs showing the distribution of CMB (white arrows) in three representative cases: **(A)** strictly deep CMB in a patient with deep left ICH, suggesting underlying hypertensive angiopathy, **(B)** mixed CMB with an acute left lobar ICH, **(C)** strictly lobar CMB associated with an acute right lobar ICH and cortical superficial siderosis (*) suggesting underlying CAA.

The MRI scans were reviewed by the investigators, blinded to all clinical data and in accordance with the Standards for Reporting Vascular Changes on Neuroimaging (STRIVE) recommendations (12). Symptomatic ICH location was categorized as either lobar (cortex or subcortical white matter) or non-lobar (thalamus, basal ganglia, brainstem or cerebellum). ICH volume was calculated using the abc/2 method (13). CMB presence and number were evaluated on the T2^*^-GRE images according to the current consensus criteria (6). They were classified as lobar (cortical and cortico-subcortical area) or deep (basal ganglia, thalamus, or brainstem). According to CMB distribution, patients were categorized as (1) strictly lobar CMB, (2) strictly deep CMB, or (3) mixed CMB (Figure 1). Two trained raters (CB and NR) independently reviewed MR images from 20 randomly selected patients. The inter-rater reliability was excellent for the CMB category (κ = 0.82). Chronic ICHs were defined as prior symptomatic or asymptomatic ICHs > 5 mm on the T2^*^-GRE images without acute bleeding identified on MRI scans. Cortical superficial siderosis (cSS) presence and extent were visually assessed according to the consensus recommended criteria (14). Periventricular and deep white matter hyperintensities (WMH) were visually assessed on axial FLAIR images on the 4-point Fazekas' rating scale (15). A total WMH score on a 7-point scale was obtained by adding up the Fazekas' scores in each region.

### Statistical Analyses

Baseline clinical and imaging data of patients with mixed CMB were compared to those with (1) strictly lobar CMB and (2) strictly deep CMB. We used χ^2^ test (or Fisher test as appropriate) for qualitative variables and Wilcoxon's rank-sum test for quantitative variables.

Multivariable logistic regression models were used to identify factors associated with mixed CMB vs. strictly lobar CMB then vs. strictly deep CMB. We categorized age as <55 vs. ≥55, and total CMB counts using cut points (0, 1, 2–4, ≥5). Potential factors were age, hypertension, lobar symptomatic ICH, total CMB count, presence of cSS, chronic lobar ICH, severe (Fazekas 5 or 6) WMH, and lacunes. Each factor was tested in a univariable model and those with *p* < 30% were inserted into multivariable models. Final models were obtained using a backward elimination strategy. All models were adjusted for age and diabetes. The final model contained only factors significant at 5%.

We calculated the ratio lobar/total CMB count (CMB ratio) to assess the predominant CMB distribution (either in lobar or deep regions) in patients with mixed CMB. We explored whether CMB ratio may indicate the underlying SVD in patients with mixed CMB by testing its association with symptomatic ICH location and cSS. Within the mixed CMB group, we compared the clinical and imaging characteristics between patients with symptomatic lobar ICH vs. non-lobar ICH. Variables with *p* < 30% in the univariable analysis were included in the multivariable model. The model was adjusted for age and diabetes. We also tested the association between CMB ratio (categorized by tertile) and both cSS and symptomatic lobar ICH using Chi^2^ or Kruskal-Wallis tests.

Statistical testing was conducted at an alpha level of 5% (two-tailed). Data were analyzed using SAS® software, version 9.4 (SAS Institute).

## Results

**Figure 2:**
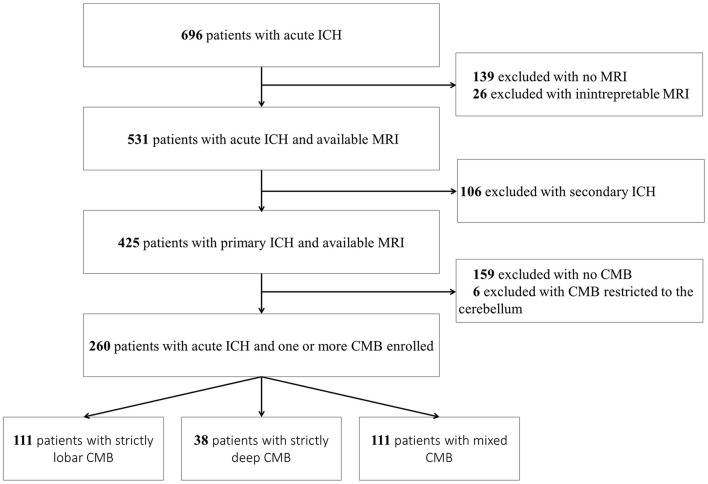
Flow diagram. CMB, cerebral microbleed; ICH, intracerebral hemorrhage.

Among 696 patients with acute non-traumatic ICH admitted to our stroke unit during the study period, 425 had a primary ICH and interpretable MRI (Figure 2). Of them, 159 [37.4%; 95% confidence interval (CI) 32.8–42.0%] had no CMB, 111 (26.1%; 95% CI 21.9–30.3%) mixed CMB, 111 (26.1%; 95% CI 21.9–30.3%) strictly lobar CMB, and 38 (8.9%; 95% CI 6.2–11.7%) had strictly deep CMB. Six patients had CMB restricted to the cerebellum and were excluded from the analysis.

**Figure 3:**
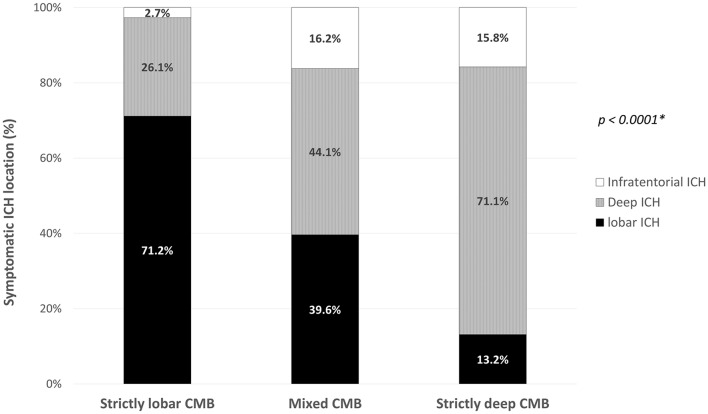
Symptomatic intracerebral hemorrhage location according to the cerebral microbleeds distribution pattern. *global χ^2^ test. CMB, cerebral microbleed; ICH, intracerebral hemorrhage.

The final cohort consisted of 260 patients with primary ICH who had ≥ 1 CMB (mean age 72.7 ± 12.2). Baseline clinical and imaging characteristics of patients with mixed CMB, strictly lobar CMB and strictly deep CMB are summarized in Table 1. Location of the symptomatic ICH varied across the three groups (*p* < 0.0001). Patients with strictly lobar CMB had predominantly lobar ICH, whereas those with strictly deep CMB were more likely to have a deep ICH (Figure 3). Presence of both lobar and non-lobar ICH (including acute and chronic ICH) was observed in 17 (15.3%) patients with mixed CMB, compared to 7 (6.3%) patients with strictly lobar CMB (*p* = 0.03) and 1 (2.6%) patient with strictly deep CMB (*p* = 0.04).

**Table 1:** Characteristics and comparison of patients with acute intracerebral hemorrhage according to cerebral microbleeds distribution.

	**Mixed CMB (*n* = 111)**	**Strictly lobar CMB (*n* = 111)**	**Strictly deep CMB (*n* = 38)**
Age, mean ± SD^*^	74 ± 9.8	73.7 ± 12.8	65.8 ± 14.9^a^
Male, No. (%)	64 (57.6)	69 (62.2)	28 (73.7)
Hypertension, No. (%)	82 (73.9)	70 (63.1)	31 (81.6)
Diabetes, No. (%)	16 (14.4)	18 (16.2)	7 (18.4)
Previous symptomatic ICH, No. (%)	9 (8.1)	11 (9.9)	2 (5.2)
Anticoagulant use, No. (%)	27 (24.3)	21 (18.9)	5 (13.2)
NIHSS at admission, median [IQR]^*^	6 [3–13]	7 [4–15]	7.5 [3–16]
Onset to MRI (days), median [IQR]^*^	1 [0–4]	1 [0–3]	1 [0–3]
Symptomatic lobar ICH, No. (%)	44 (39.6)	79 (71.2)^b^	5 (13.2)^a^
Presence of cSS, No. (%)	27 (24.3)	49 (44.1)^a^	4 (10.5)
Presence of Disseminated cSS, No. (%)	15 (13.5)	27 (24.3)^a^	1 (2.6)
CMB count, median [IQR]^*^	11 [6–27]	3 [1–9]^b^	2 [1–4]^b^
Presence of chronic lobar ICH, No. (%)	27 (24.3)	23 (20.7)	1 (2.6)^a^
Fazekas' WMH score/6, median [IQR]^*^	4 [3–6]	3 [2–4]^b^	3 [2–5]^b^
Presence of lacune, No. (%)	72 (64.8)	31 (27.9)^b^	16 (42.1)^a^

*CMB, cerebral microbleed; cSS, cortical superficial siderosis; ICH, intracerebral hemorrhage; NIHSS, National Institute of Health Stroke Score; WMH, white matter hyperintensities*.

*The p-values reported in the strictly lobar CMB column refer to comparison with mixed CMB. The p-values reported in the strictly deep CMB column refer to comparison with mixed CMB*.

p-values were obtained via χ^2^ test or Wilcoxon's rank-sum test (*).

a *p < 0.05*;

b *p < 0.001*.

### Mixed CMB Compared to Strictly Lobar CMB

Compared to patients with strictly lobar CMB, patients with mixed CMB were similar in age and tended to have more frequent history of hypertension (*p* = 0.08). The symptomatic ICH was lobar in 44 (39.6%) patients with mixed CMB compared to 79 (71.2%) patients with strictly lobar CMB (*p* < 0.0001). Patients with mixed CMB were more likely to have lacunes and had a more severe burden of CMB and WMH than those with strictly lobar CMB. Conversely, cSS was more common in patients with strictly lobar CMB than mixed CMB (44.1 vs. 24.3%; *p* < 0.002). Multivariable model shows that compared to strictly lobar CMB, mixed CMB pattern was associated with the presence of severe WMH [odds ratio (OR) 3.38, 95% CI 1.52–7.80, *p* = 0.003], lacunes (OR 3.77, 95% CI 1.83–8.03, *p* = 0.0004), and higher CMB count (OR 7.40, 95% CI 3.96–15.10, *p* < 0.0001). Conversely, strictly lobar CMB pattern was associated with symptomatic lobar ICH (OR 4.71, 95% CI 2.16–10.96, *p* = 0.0002) and cSS (OR 3.09, 95% CI 1.40–7.04, *p* = 0.006).

### Mixed CMB Compared to Strictly Deep CMB

Compared to patients with strictly deep CMB, patients with mixed CMB were older but had similar prevalence of hypertension and diabetes. Patients with mixed CMB were more likely to have a symptomatic lobar ICH than those with strictly deep CMB and had a more severe burden of lacunes, CMB, and WMH. Prevalence of cSS did not differ between the two groups. A multivariable model shows that compared to strictly deep CMBs, the pattern of mixed CMB was associated with symptomatic lobar ICH (OR 4.68, 95%, CI 1.27–21.68, *p* = 0.03) and higher CMB count (OR 11.69, 95% CI 5.43–29.62, *p* < 0.0001).

### Mixed CMB, ICH Location, and Cortical Superficial Siderosis

Among patients with mixed CMB, 44 (39.6%) had a lobar symptomatic ICH and 67 (60.4%) had a non-lobar ICH. The two groups were similar in age (Table 2). Patients with non-lobar ICH and mixed CMB were more likely to be male and to have hypertension, whereas patients with lobar ICH and mixed CMB were more likely to have cSS and chronic lobar ICH. Although total CMB count was similar between the two groups, patients with lobar ICH had higher lobar CMB counts, whereas patients with non-lobar ICH had higher deep CMB counts. Among patients with mixed CMB, the ratio lobar CMB count/total CMB count was higher in patients with lobar ICH than those with non-lobar ICH. In the multivariable analysis, non-lobar symptomatic ICH location was associated with male gender (OR 3.44 95%, CI 1.28–9.91, *p* = 0.02), history of hypertension (OR 7.41, 95% CI 2.57–23.74, *p* = 0.0004), and higher deep CMB counts (OR 2.59, 95% CI 1.50–4.80, *p* = 0.001), whereas lobar ICH location was associated with cSS (OR 3.43, 95% CI 1.18–10.54, *p* = 0.03).

**Table 2:** Comparison between patients with lobar and non-lobar symptomatic ICH in subjects with mixed cerebral microbleeds.

	**Mixed CMB**		***p*-value**
	**Lobar ICH**	**Non-lobar ICH**	
No. (%)	44 (39.6)	67 (60.4)	–
**Clinical characteristics**			
Age, mean ± SD	75.4 ± 9.0	73.0 ± 10.2	0.30^*^
Male, No. (%)	19 (43.2)	45 (67.2)	0.01
Hypertension, No. (%)	24 (54.6)	58 (86.6)	0.0002
Diabetes, No. (%)	5 (11.4)	11 (16.4)	0.46
**Imaging characteristics**			
Presence of cSS, No. (%)	17 (38.6)	10 (14.9)	0.004
CMB count, median [IQR]	13 [6–39]	10 [5–24]	0.17^*^
Lobar CMB count, median [IQR]	10.5 [3–28.5]	4 [2–13]	0.004^*^
Deep CMB count, median [IQR]	1.5 [1–4]	3 [2–6]	0.008^*^
Ratio lobar/total CMB, median [IQR]	0.8 [0.5–0.9]	0.5 [0.3–0.6]	<0.0001^*^
Presence of chronic lobar ICH, No. (%)	15 (34.1)	12 (17.9)	0.05
Severe WMH (Fazekas 5–6), No. (%)	20 (45.5)	30 (44.8)	0.94
Presence of lacune, No. (%)	24 (54.6)	48 (71.6)	0.07

*CMB, cerebral microbleed; cSS, cortical superficial siderosis; ICH, intracerebral hemorrhage; WMH, white matter hyperintensities*.

p-values were obtained via χ^2^ test or Wilcoxon's rank-sum test (*).

**Figure 4:**
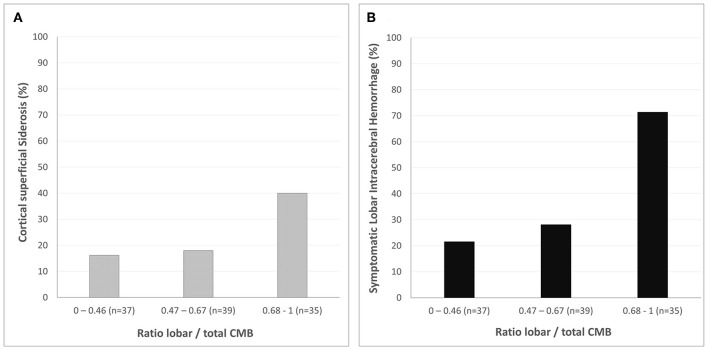
Cerebral microbleeds distribution and markers of CAA in patients with mixed cerebral microbleeds. Prevalence of cortical superficial siderosis **(A)** and symptomatic lobar intracerebral hemorrhage **(B)** according to the ratio lobar/total CMB count (categorized by tertile). Increasing cerebral microbleeds ratio is associated with both cortical superficial siderosis (*p* = 0.03) and lobar intracerebral hemorrhage (*p* < 0.0001).

Increasing CMB ratio was associated with increasing prevalence of cSS (*p* = 0.03) and symptomatic lobar ICH (*p* < 0.0001) in patients with mixed CMB (Figure 4).

## Discussion

Our study shows that mixed CMB is frequently encountered in subjects with symptomatic ICH and might be due to different degrees of both CAA and HA. Approximatively 25% of patients with mixed CMB have cSS and may therefore harbor CAA, as dominant or coexisting underlying SVD. Along with cSS, ICH location and the pattern of CMB distribution may indicate the predominant underlying vasculopathy in patients with mixed CMB.

In our large cohort of patients with primary ICH, 26% had mixed CMB, confirming that mixed hemorrhage distribution is a common condition in adults with symptomatic ICH (16–20). In accordance with previous studies (18), we found that mixed CMB was associated with a more severe burden of SVD including lacunes, CMB, and WMH, compared to patients with strictly lobar and strictly deep CMB. Interestingly, in our cohort, prevalence of hypertension and diabetes was similar between patients with mixed CMB and the two other groups. The increased severity of SVD associated with mixed CMB may not therefore be driven by vascular risk factors alone.

Whether patients with mixed CMB have advanced HA, CAA, or both is still debated. As both CAA and hypertension are common in the elderly (21, 22), concomitant CAA and HA is a possible hypothesis. Based on post-mortem studies showing that up to 15% of patients with deep ICH had pathological evidence of CAA (23), it has been hypothesized that most patients with mixed CMB would have both CAA and HA. However, it has also been shown that HA could promote lobar CMB, suggesting that patients with mixed CMB may have advanced HA as underlying SVD (8). Results from a recent large imaging study support this hypothesis, showing that patients with mixed ICH are more similar to HA-ICH than CAA-ICH (18).

In our cohort, mixed CMB appears as a relatively heterogeneous group with different ICH subtypes. Most patients with mixed CMB presented with a non-lobar symptomatic ICH and high prevalence of hypertension, suggesting HA as predominant underlying SVD. Nevertheless, cSS was observed in 39% of patients with mixed CMB presenting with a lobar symptomatic ICH, suggesting that a substantial proportion of these patients may have some degree of CAA. A recent amyloid PET study in an Asian ICH population supports this hypothesis (24). Although patients with mixed ICH had overall low burden of amyloid PET, a small subset with mixed ICH who had cSS showed similar high burden of amyloid PET than CAA-ICH.

Interestingly, CMB distribution in patients with mixed CMB may differ according to the ICH location and the presence of cSS. Increasing lobar/total CMB ratio was associated with symptomatic lobar ICH and cSS, two key neuroimaging markers of CAA. Hence, the ratio lobar/total CMB may be an interesting biomarker that could help to identify underlying CAA in patients with mixed CMB and should be assessed along with cSS.

Our study has limitations, including its retrospective and MRI-based design. This could have led to a selection bias toward the less severe ICH cases, but nonetheless 76% of patients screened for this study underwent MRI and were included in the analysis. Moreover, unlike previous studies (16–20), our “mixed” category referred to mixed CMB instead of mixed hemorrhage (i.e., including symptomatic ICH and CMB). Therefore, our findings are not entirely comparable to these previous reports. However, we designed our study to assess lobar ICH, along with cSS, as imaging marker of CAA (10). In a clinical setting, patients underwent either 1.5-T or 3-T MRI. This could have affected our CMB detection. However, we did not find any difference in MRI field strength between the three groups. Finally, the most important limitation is the lack of pathological examinations. Although patients were subject to rigorous selection criteria to limit diagnostic uncertainty, further studies with MRI and pathological validation are needed to confirm our findings.

In summary, mixed CMB is a frequent condition, observed in ~25% of patients with primary ICH, characterized by a severe burden of SVD. Mixed CMB appears as a heterogenous vasculopathy that may be due to different degrees of both HA and CAA. Although most subjects with mixed CMB seem to have HA as dominant underlying SVD, 25% of patients with mixed CMB have cSS, suggesting dominant or coexisting CAA. Along with cSS and lobar ICH, a predominantly lobar CMB distribution (assessed by the ratio lobar/total CMB) may be a useful biomarker to identify underlying CAA in these patients. As CAA is associated with an increased risk of recurrent ICH, these findings may have important prognostic implications.

## Data Availability Statement

The datasets generated for this study are available on request to the corresponding author.

## Ethics Statement

The studies involving human participants were reviewed and approved by Toulouse-Purpan Hospital Research Ethics Committee. Written informed consent for participation was not required for this study in accordance with the national legislation and the institutional requirements.

## Author Contributions

NR: conception, design, and supervision. VR: statistical analyses. CB and NR: data collection, writing, and tables and figures. All authors: data analyses and interpretation, review and editing, and read and approved the final manuscript.

### Conflict of Interest

The authors declare that the research was conducted in the absence of any commercial or financial relationships that could be construed as a potential conflict of interest.
